# 6β-Hy­droxy­eremophil-7(11)-en-8β,12-olide

**DOI:** 10.1107/S1600536811016308

**Published:** 2011-05-07

**Authors:** Ri-Na Su, Sha Shi, Hai-Bo Wu, Wen-Shu Wang

**Affiliations:** a College of Life and Environment Science, Minzu University of China, Beijing 100081, People’s Republic of China

## Abstract

The title eremophilenolide, C_15_H_22_O_3_, is a natural compound isolated from *Senecio laetus* Edgew. The two *cis*-fused six-membered rings have chair confomations and the five-membered ring has a planar envelope conformation [maximum deviation = 0.010 (1) Å]. The β-hy­droxy group participates in inter­molecular O—H⋯O hydrogen bonding, forming mol­ecular chains along the *a* axis.

## Related literature

For related compounds extracted from *Ligularia fischeri* and *Ligularia duciformis*, see: Wang *et al.* (2000 ▶) and Fu *et al.* (2007 ▶), respectively.
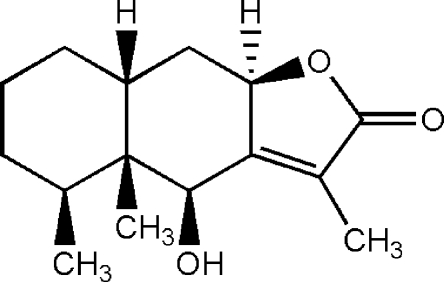


## Experimental

### 

#### Crystal data

C_15_H_22_O_3_
*M* *_r_* = 250.33
Orthorhombic, 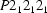

*a* = 8.0141 (16) Å
*b* = 9.969 (2) Å
*c* = 16.482 (3) Å
*V* = 1316.8 (5) Å^3^
*Z* = 4
Mo *K*α radiation
μ = 0.09 mm^−1^
*T* = 293 K
0.60 × 0.60 × 0.30 mm

#### Data collection

Rigaku R-AXIS RAPID IP diffractometer
Absorption correction: multi-scan (*ABSCOR*; Higashi, 1995 ▶) *T*_min_ = 0.950, *T*_max_ = 0.975
8965 measured reflections
1744 independent reflections
1314 reflections with *I* > 2σ(*I*)
*R*_int_ = 0.061

#### Refinement

*R*[*F* ^2^ > 2σ(*F* ^2^)] = 0.047
*wR*(*F* ^2^) = 0.107
*S* = 0.95
1744 reflections
168 parameters
H-atom parameters constrained
Δρ_max_ = 0.21 e Å^−3^
Δρ_min_ = −0.20 e Å^−3^

### 

Data collection: *RAPID-AUTO* (Rigaku, 2000 ▶); cell refinement: *RAPID-AUTO*; data reduction: *CrystalStructure* (Rigaku/MSC, 2000 ▶); program(s) used to solve structure: *SHELXS97* (Sheldrick, 2008 ▶); program(s) used to refine structure: *SHELXL97* (Sheldrick, 2008 ▶); molecular graphics: *SHELXTL* (Sheldrick, 2008 ▶); software used to prepare material for publication: *SHELXL97*.

## Supplementary Material

Crystal structure: contains datablocks I, global. DOI: 10.1107/S1600536811016308/ld2005sup1.cif

Structure factors: contains datablocks I. DOI: 10.1107/S1600536811016308/ld2005Isup2.hkl

Additional supplementary materials:  crystallographic information; 3D view; checkCIF report

## Figures and Tables

**Table 1:** Hydrogen-bond geometry (Å, °)

*D*—H⋯*A*	*D*—H	H⋯*A*	*D*⋯*A*	*D*—H⋯*A*
O3—H3⋯O1^i^	0.82	2.18	2.931 (2)	152

Symmetry code: (i) 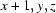.

**Fig. 1.**
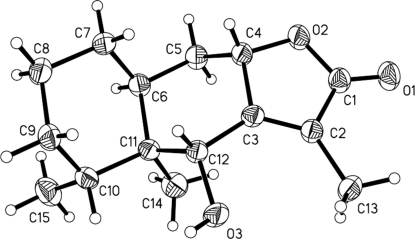
View of the title molecule showing the atom-numbering scheme. Displacement ellipsoids are drawn at the 50% probability level.

## supplementary crystallographic information

### Comment

*Senecio laetus* Edgew. grows in Guizhou province of China, and is traditionally used by the locals as medicine having effects on clearing fever and detoxifcation and relieving cough activities. As a part of our research on biological resources in the western area in China, the title compound, an eremophilenolide, was isolated. The compound was identified by NMR spectra, which were compared with the previous reports (Wang *et al.*, 2000; Fu *et al.*, 2007). Herewith, we present its crystal structure. The molecule consists of a fused three-ring system A/B/C(Fig.1). The rings A(C10–C6/C11)and B(C6–C3/C12–C11) are *cis*-fused; the hydroxy group at C12 site has the same β-orientation as the two methyl groups at C10 and C11. Rings A and B both are in chair conformations. The lactone ring has an envelope-like conformation with the atoms C1-C4 and O2 deviating from its mean plane by -0.002 (1), 0.008 (1), -0.010 (1), 0.007 (1) and -0.003 (1), respectively. The inter-molecular O—H···O hydrogen bonds apparently stabilize the crystal structure linking molecules into chains along [1 0 0].

### Experimental

The air-dried whole plants of *Senecio laetus* (0.7 kg) were pulverized and triply extracted with MeOH (each time for 7 days) at room temperature. The extract was concentrated to give a residue (67 g), which was further separated by CC (SiO_2_, 200–300mesh, petroleum ether/EtOAc (20:1, 15:1, 10:1, 8:1, 5:1, 3:1, 2:1, 1:1 (*v*/*v*)) to yield 8 fractions: Fr. 1–8. Each fraction was examined by TLC and combined to afford many subfractions. Fr.5a (0.5 g) was subjected to CC (SiO_2_, 200–300mesh, petroleum ether/ EtOAc 10:1, 5:1 (*v*/*v*)) to provide the title compound (20 mg). ^1^H and ^13^C NMR spectral data of this compound was recorded on Bruker-AV-500 s pectrometer, using CDCl_3_ as solvent and Me_4_Si as internal standard. The stereochemistry was established by the X-ray diffraction experiment.

### Refinement

The hydrogen atoms were placed in calculated positions [d(O—H) = 0.82 Å] and refined as riding with *U*_iso_(H) = 1.2Ueq(C) or 1.5Ueq(C,*O*). The positions of methyl and hydroxy hydrogens were rotationally optimized. In the absence of any significant anomalous scatterers in the molecule, the absolute configuration has been arbitrarily assigned. Friedel pairs have been averaged.

### Crystal data

**Table:**

C_15_H_22_O_3_	*F*(000) = 544
*M**_r_* = 250.33	*D*_x_ = 1.263 Mg m^−^^3^
Orthorhombic, *P*2_1_2_1_2_1_	Mo *K*α radiation, λ = 0.71073 Å
Hall symbol: P 2ac 2ab	Cell parameters from 8965 reflections
*a* = 8.0141 (16) Å	θ = 2.4–27.5°
*b* = 9.969 (2) Å	µ = 0.09 mm^−^^1^
*c* = 16.482 (3) Å	*T* = 293 K
*V* = 1316.8 (5) Å^3^	Block, colourless
*Z* = 4	0.60 × 0.60 × 0.30 mm

### Data collection

**Table:**

Rigaku R-AXIS RAPID IP diffractometer	1744 independent reflections
Radiation source: fine-focus sealed tube	1314 reflections with *I* > 2σ(*I*)
graphite	*R*_int_ = 0.061
ω scans	θ_max_ = 27.5°, θ_min_ = 2.4°
Absorption correction: multi-scan (*ABSCOR*; Higashi, 1995)	*h* = 0→10
*T*_min_ = 0.950, *T*_max_ = 0.975	*k* = 0→12
8965 measured reflections	*l* = 0→21

### Refinement

**Table:**

Refinement on *F*^2^	Secondary atom site location: difference Fourier map
Least-squares matrix: full	Hydrogen site location: inferred from neighbouring sites
*R*[*F*^2^ > 2σ(*F*^2^)] = 0.047	H-atom parameters constrained
*wR*(*F*^2^) = 0.107	*w* = 1/[σ^2^(*F*_o_^2^) + (0.0635*P*)^2^] where *P* = (*F*_o_^2^ + 2*F*_c_^2^)/3
*S* = 0.95	(Δ/σ)_max_ = 0.002
1744 reflections	Δρ_max_ = 0.21 e Å^−^^3^
168 parameters	Δρ_min_ = −0.20 e Å^−^^3^
0 restraints	Extinction correction: *SHELXL97* (Sheldrick, 2008), Fc^*^=kFc[1+0.001xFc^2^λ^3^/sin(2θ)]^-1/4^
Primary atom site location: structure-invariant direct methods	Extinction coefficient: 0.180 (11)

### Special details

**Table:**

Experimental. Since the two skeleton methyl groups in eremophilenolides are in biogenic β orientation, we assigned the relative stereochemistry of the title eremophilenolide, by reference to the structures of related eremophilenolides in Wang *et al.* (2000) and Fu *et al.* (2007) although the absolute configuration could not be reliably determined from anomalous dispersion effects with Mo radiation used in the experiment. Furthermore, the relative stereochemistry in the title compound was confirmed by NMR data. ^13^C NMR(125 MHz, CDCl_3_, δ, p.p.m.): 175.15(C1), 162.95(C3), 122.08(C2), 77.55(C4), 70.18(C12), 45.60(C11), 35.88(C5), 35.31(C6), 31.68(C10), 28.33(C7), 28.33(C9), 20.17(C8), 18.96(C14), 15.14(C15), 8.93(C13).
Geometry. All e.s.d.'s (except the e.s.d. in the dihedral angle between two l.s. planes) are estimated using the full covariance matrix. The cell e.s.d.'s are taken into account individually in the estimation of e.s.d.'s in distances, angles and torsion angles; correlations between e.s.d.'s in cell parameters are only used when they are defined by crystal symmetry. An approximate (isotropic) treatment of cell e.s.d.'s is used for estimating e.s.d.'s involving l.s. planes.
Refinement. Refinement of *F*^2^ against ALL reflections. The weighted *R*-factor *wR* and goodness of fit *S* are based on *F*^2^, conventional *R*-factors *R* are based on *F*, with *F* set to zero for negative *F*^2^. The threshold expression of *F*^2^ > σ(*F*^2^) is used only for calculating *R*-factors(gt) *etc.* and is not relevant to the choice of reflections for refinement. *R*-factors based on *F*^2^ are statistically about twice as large as those based on *F*, and *R*- factors based on ALL data will be even larger.

### Fractional atomic coordinates and isotropic or equivalent isotropic displacement parameters (Å^2^)

**Table:**

	*x*	*y*	*z*	*U*_iso_*/*U*_eq_	
C1	0.1478 (3)	1.0934 (2)	−0.03828 (13)	0.0498 (5)	
C2	0.3268 (3)	1.0825 (2)	−0.05855 (13)	0.0440 (5)	
C3	0.4112 (3)	1.0805 (2)	0.01139 (12)	0.0413 (5)	
C4	0.2914 (2)	1.0938 (3)	0.08081 (12)	0.0481 (6)	
H4	0.3130	1.1778	0.1099	0.058*	
C5	0.3057 (3)	0.9777 (3)	0.13851 (14)	0.0539 (6)	
H5A	0.2681	0.8962	0.1120	0.065*	
H5B	0.2354	0.9933	0.1854	0.065*	
C6	0.4879 (3)	0.9612 (3)	0.16573 (12)	0.0468 (5)	
H6	0.4924	0.8796	0.1989	0.056*	
C7	0.5409 (3)	1.0768 (3)	0.22055 (13)	0.0549 (6)	
H7A	0.4673	1.0804	0.2672	0.066*	
H7B	0.5293	1.1606	0.1911	0.066*	
C8	0.7215 (3)	1.0626 (3)	0.24987 (13)	0.0614 (7)	
H8A	0.7529	1.1411	0.2811	0.074*	
H8B	0.7314	0.9845	0.2846	0.074*	
C9	0.8371 (3)	1.0479 (3)	0.17726 (13)	0.0550 (6)	
H9A	0.9506	1.0361	0.1965	0.066*	
H9B	0.8336	1.1297	0.1455	0.066*	
C10	0.7906 (3)	0.9293 (2)	0.12286 (13)	0.0497 (6)	
H10	0.8622	0.9345	0.0748	0.060*	
C11	0.6069 (2)	0.9379 (2)	0.09238 (12)	0.0427 (5)	
C12	0.5903 (2)	1.0574 (2)	0.03156 (12)	0.0413 (5)	
H12	0.6343	1.1384	0.0576	0.050*	
C13	0.3809 (3)	1.0747 (3)	−0.14545 (12)	0.0561 (6)	
H13A	0.4807	1.1265	−0.1528	0.084*	
H13B	0.2943	1.1096	−0.1797	0.084*	
H13C	0.4024	0.9829	−0.1596	0.084*	
C14	0.5597 (3)	0.8095 (2)	0.04675 (15)	0.0633 (7)	
H14A	0.6471	0.7864	0.0095	0.095*	
H14B	0.4578	0.8239	0.0173	0.095*	
H14C	0.5444	0.7377	0.0848	0.095*	
C15	0.8317 (4)	0.7963 (3)	0.16557 (18)	0.0727 (8)	
H15A	0.9469	0.7961	0.1815	0.109*	
H15B	0.8113	0.7230	0.1291	0.109*	
H15C	0.7625	0.7867	0.2128	0.109*	
O1	0.0277 (2)	1.09467 (19)	−0.08281 (10)	0.0629 (5)	
O2	0.12877 (17)	1.09976 (19)	0.04302 (9)	0.0568 (5)	
O3	0.67936 (19)	1.0359 (2)	−0.04189 (9)	0.0647 (6)	
H3	0.7767	1.0594	−0.0360	0.097*	

### Atomic displacement parameters (Å^2^)

**Table:**

	*U*^11^	*U*^22^	*U*^33^	*U*^12^	*U*^13^	*U*^23^
C1	0.0432 (11)	0.0585 (13)	0.0476 (11)	−0.0017 (11)	−0.0011 (10)	0.0074 (12)
C2	0.0401 (10)	0.0499 (12)	0.0420 (10)	−0.0017 (10)	−0.0004 (9)	0.0037 (10)
C3	0.0377 (9)	0.0479 (11)	0.0383 (9)	−0.0040 (10)	0.0045 (9)	0.0014 (9)
C4	0.0310 (9)	0.0733 (15)	0.0399 (10)	0.0022 (11)	0.0015 (9)	0.0018 (11)
C5	0.0352 (10)	0.0824 (17)	0.0441 (11)	−0.0036 (12)	0.0082 (9)	0.0135 (12)
C6	0.0361 (10)	0.0641 (13)	0.0403 (10)	−0.0009 (10)	0.0069 (9)	0.0113 (10)
C7	0.0448 (11)	0.0820 (16)	0.0378 (10)	0.0094 (13)	0.0056 (10)	−0.0024 (11)
C8	0.0484 (12)	0.0904 (19)	0.0455 (11)	−0.0002 (14)	−0.0053 (11)	−0.0075 (13)
C9	0.0384 (11)	0.0781 (16)	0.0486 (11)	−0.0010 (12)	−0.0044 (11)	−0.0014 (12)
C10	0.0381 (11)	0.0629 (14)	0.0481 (11)	0.0058 (11)	0.0048 (10)	0.0028 (11)
C11	0.0356 (10)	0.0507 (12)	0.0418 (10)	−0.0012 (9)	0.0024 (9)	−0.0009 (9)
C12	0.0327 (9)	0.0556 (12)	0.0357 (9)	−0.0045 (9)	0.0031 (8)	0.0033 (10)
C13	0.0574 (14)	0.0724 (15)	0.0386 (10)	−0.0003 (13)	0.0000 (11)	0.0016 (11)
C14	0.0605 (14)	0.0576 (13)	0.0717 (15)	−0.0026 (12)	−0.0018 (14)	−0.0084 (13)
C15	0.0631 (16)	0.0726 (16)	0.0825 (17)	0.0173 (14)	−0.0060 (16)	0.0115 (15)
O1	0.0436 (8)	0.0838 (12)	0.0612 (9)	−0.0014 (9)	−0.0117 (8)	0.0080 (9)
O2	0.0333 (7)	0.0884 (12)	0.0488 (8)	0.0062 (9)	0.0030 (7)	0.0099 (9)
O3	0.0375 (8)	0.1178 (16)	0.0388 (7)	−0.0027 (10)	0.0079 (7)	−0.0008 (10)

### Geometric parameters (Å, °)

**Table:**

C1—O1	1.210 (3)	C8—H8B	0.9700
C1—O2	1.350 (3)	C9—C10	1.530 (3)
C1—C2	1.477 (3)	C9—H9A	0.9700
C2—C3	1.337 (3)	C9—H9B	0.9700
C2—C13	1.499 (3)	C10—C15	1.537 (3)
C3—C12	1.492 (3)	C10—C11	1.557 (3)
C3—C4	1.500 (3)	C10—H10	0.9800
C4—O2	1.445 (2)	C11—C14	1.532 (3)
C4—C5	1.502 (3)	C11—C12	1.563 (3)
C4—H4	0.9800	C12—O3	1.421 (2)
C5—C6	1.536 (3)	C12—H12	0.9800
C5—H5A	0.9700	C13—H13A	0.9600
C5—H5B	0.9700	C13—H13B	0.9600
C6—C7	1.525 (4)	C13—H13C	0.9600
C6—C11	1.557 (3)	C14—H14A	0.9600
C6—H6	0.9800	C14—H14B	0.9600
C7—C8	1.532 (3)	C14—H14C	0.9600
C7—H7A	0.9700	C15—H15A	0.9600
C7—H7B	0.9700	C15—H15B	0.9600
C8—C9	1.521 (3)	C15—H15C	0.9600
C8—H8A	0.9700	O3—H3	0.8200
			
O1—C1—O2	120.8 (2)	C8—C9—H9B	109.0
O1—C1—C2	129.5 (2)	C10—C9—H9B	109.0
O2—C1—C2	109.71 (19)	H9A—C9—H9B	107.8
C3—C2—C1	107.30 (19)	C9—C10—C15	110.24 (19)
C3—C2—C13	132.62 (19)	C9—C10—C11	112.15 (19)
C1—C2—C13	120.07 (19)	C15—C10—C11	113.4 (2)
C2—C3—C12	132.96 (19)	C9—C10—H10	106.9
C2—C3—C4	109.45 (18)	C15—C10—H10	106.9
C12—C3—C4	117.38 (17)	C11—C10—H10	106.9
O2—C4—C3	104.59 (16)	C14—C11—C6	110.74 (18)
O2—C4—C5	111.93 (18)	C14—C11—C10	110.26 (19)
C3—C4—C5	111.4 (2)	C6—C11—C10	109.67 (16)
O2—C4—H4	109.6	C14—C11—C12	107.52 (16)
C3—C4—H4	109.6	C6—C11—C12	109.39 (17)
C5—C4—H4	109.6	C10—C11—C12	109.22 (17)
C4—C5—C6	109.90 (18)	O3—C12—C3	108.46 (17)
C4—C5—H5A	109.7	O3—C12—C11	112.88 (18)
C6—C5—H5A	109.7	C3—C12—C11	110.02 (16)
C4—C5—H5B	109.7	O3—C12—H12	108.5
C6—C5—H5B	109.7	C3—C12—H12	108.5
H5A—C5—H5B	108.2	C11—C12—H12	108.5
C7—C6—C5	110.9 (2)	C2—C13—H13A	109.5
C7—C6—C11	113.71 (18)	C2—C13—H13B	109.5
C5—C6—C11	111.80 (17)	H13A—C13—H13B	109.5
C7—C6—H6	106.7	C2—C13—H13C	109.5
C5—C6—H6	106.7	H13A—C13—H13C	109.5
C11—C6—H6	106.7	H13B—C13—H13C	109.5
C6—C7—C8	112.3 (2)	C11—C14—H14A	109.5
C6—C7—H7A	109.1	C11—C14—H14B	109.5
C8—C7—H7A	109.1	H14A—C14—H14B	109.5
C6—C7—H7B	109.1	C11—C14—H14C	109.5
C8—C7—H7B	109.1	H14A—C14—H14C	109.5
H7A—C7—H7B	107.9	H14B—C14—H14C	109.5
C9—C8—C7	109.66 (18)	C10—C15—H15A	109.5
C9—C8—H8A	109.7	C10—C15—H15B	109.5
C7—C8—H8A	109.7	H15A—C15—H15B	109.5
C9—C8—H8B	109.7	C10—C15—H15C	109.5
C7—C8—H8B	109.7	H15A—C15—H15C	109.5
H8A—C8—H8B	108.2	H15B—C15—H15C	109.5
C8—C9—C10	112.8 (2)	C1—O2—C4	108.92 (16)
C8—C9—H9A	109.0	C12—O3—H3	109.5
C10—C9—H9A	109.0		
			
O1—C1—C2—C3	−177.7 (3)	C7—C6—C11—C10	50.5 (2)
O2—C1—C2—C3	1.0 (3)	C5—C6—C11—C10	177.0 (2)
O1—C1—C2—C13	1.6 (4)	C7—C6—C11—C12	−69.2 (2)
O2—C1—C2—C13	−179.7 (2)	C5—C6—C11—C12	57.3 (2)
C1—C2—C3—C12	172.8 (2)	C9—C10—C11—C14	−173.30 (18)
C13—C2—C3—C12	−6.4 (4)	C15—C10—C11—C14	−47.6 (3)
C1—C2—C3—C4	−1.6 (3)	C9—C10—C11—C6	−51.1 (2)
C13—C2—C3—C4	179.2 (3)	C15—C10—C11—C6	74.6 (3)
C2—C3—C4—O2	1.6 (3)	C9—C10—C11—C12	68.8 (2)
C12—C3—C4—O2	−173.75 (18)	C15—C10—C11—C12	−165.52 (19)
C2—C3—C4—C5	122.8 (2)	C2—C3—C12—O3	0.9 (3)
C12—C3—C4—C5	−52.6 (3)	C4—C3—C12—O3	174.9 (2)
O2—C4—C5—C6	170.32 (18)	C2—C3—C12—C11	−123.0 (3)
C3—C4—C5—C6	53.6 (3)	C4—C3—C12—C11	51.0 (3)
C4—C5—C6—C7	69.5 (2)	C14—C11—C12—O3	−52.3 (2)
C4—C5—C6—C11	−58.5 (3)	C6—C11—C12—O3	−172.67 (16)
C5—C6—C7—C8	179.21 (18)	C10—C11—C12—O3	67.3 (2)
C11—C6—C7—C8	−53.8 (3)	C14—C11—C12—C3	69.0 (2)
C6—C7—C8—C9	55.2 (3)	C6—C11—C12—C3	−51.4 (2)
C7—C8—C9—C10	−56.9 (3)	C10—C11—C12—C3	−171.41 (18)
C8—C9—C10—C15	−71.1 (3)	O1—C1—O2—C4	178.9 (2)
C8—C9—C10—C11	56.3 (3)	C2—C1—O2—C4	0.1 (3)
C7—C6—C11—C14	172.43 (19)	C3—C4—O2—C1	−1.0 (3)
C5—C6—C11—C14	−61.0 (3)	C5—C4—O2—C1	−121.8 (2)

### Hydrogen-bond geometry (Å, °)

**Table:**

*D*—H···*A*	*D*—H	H···*A*	*D*···*A*	*D*—H···*A*
O3—H3···O1^i^	0.82	2.18	2.931 (2)	152

Symmetry codes: (i) *x*+1, *y*, *z*.
